# Direct Graphene Deposition via a Modified Laser-Assisted Method for Interdigitated Microflexible Supercapacitors

**DOI:** 10.1021/acsanm.3c05387

**Published:** 2024-02-09

**Authors:** Nikolaos Samartzis, Michail Athanasiou, Labrini Sygellou, Spyros N. Yannopoulos

**Affiliations:** † Foundation for Research and Technology Hellas, Institute of Chemical Engineering Sciences (FORTH/ICE-HT), Patras GR-26504, Greece; ‡ Department of Physics, University of Patras, Patras GR-26504, Greece; § Department of Chemistry, University of Patras, Patras GR-26504, Greece

**Keywords:** laser-induced graphene, graphene synthesis, graphene transfer, interdigitated supercapacitors, flexible supercapacitors

## Abstract

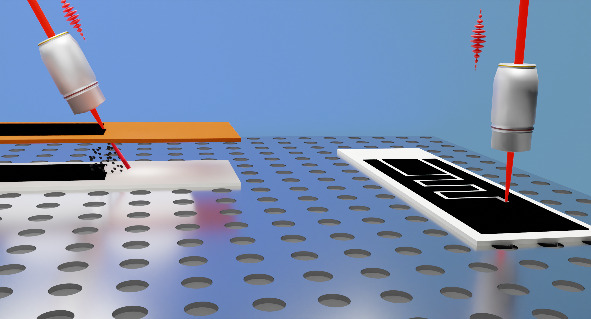

The transcendence toward smarter technologies and the rapid expansion of the Internet of Things requires miniaturized energy storage systems, which may also be shape-conformable, such as microflexible supercapacitors. Their fabrication must be compatible with emerging manufacturing platforms with regard to scalability and sustainability. Here, we modify a laser-based method we recently developed for simultaneously synthesizing and transferring graphene onto a selected substrate. The modification of the method lies in the tuning of two key parameters, namely, the inclination of the laser beam and the distance between the precursor material and the acceptor substrate. A proper combination of these parameters enables the displacement of the trace of the transmitted laser beam from the deposited graphene film area. This mitigates the negative effects that arise from the laser-induced ablation of graphene on heat-sensitive substrates and significantly improves the electrical conductivity of the graphene films. The optimized graphene exhibits very high C/O (36) and sp^2^/sp^3^ (13) ratios. Post-transport irradiation was used to transform the continuous graphene films to interdigitated electrodes. The capacitance of the microflexible supercapacitor was measured to be among the highest reported ones in relation to interdigitated supercapacitors with electrodes based on laser-grown graphene. The device shows good cycling stability, retaining 91% of its capacitance after 10,000 cycles, showing no substantial degradation after applying bending conditions. This promising laser-based approach emerges as a viable alternative for the fabrication of microflexible interdigitated supercapacitors for paper electronics and smart textiles.

## 1 Introduction

We witness nowadays a booming increase in the number of low-energy demanding wearable devices that constitute an essential part of the Internet of Things (IoT), the network of physical objects that receive and transmit data. Such flexible electronic devices persistently carried by the human body can provide vital information about body function and environmental changes, acting as an interface between the user and the surroundings. Along with the need for developing a sustainable power supply of such wearables, the fabrication of small and compact miniaturized electrochemical energy storage (EES) devices is also highly demanded for advancing smart textile applications.^1^ For their successful integration, EES devices should fulfill certain criteria concerning their mechanical robustness, flexibility and stretchability, thinness and lightweightness, and at the same time, safe and long-lasting, maintaining adequate energy and power density.^2−4^ Among the various alternative designs, flexible in-plane supercapacitors have shown high potential to fulfill the above requirements not only because they can satisfy the demands in terms of shape conformability but also as they could bridge the energy/power density gap between batteries and conventional capacitors.

Since the early development of electrochemical energy storage devices, carbon-based materials (such as hard carbons, carbon nanotubes, graphene-like networks, etc.) have been considered to be among the most promising active materials, owing to the abundance and low cost of carbon, its high conductivity and lightweightness, and the feasibility to prepare porous carbons scaffolds with extremely high microporosity.^5−7^ While carbon-based materials have been extensively studied as active materials in flexible supercapacitors,^8−10^ the fabrication methods have up until now been laborious, energy-intensive, and environmentally unfriendly. Therefore, it is still necessary to explore and establish alternative synthesis protocols that are ecofriendly, inexpensive, simple, and compatible with additive manufacturing processes to achieve direct integration of microflexible EES into the relevant products.

During the past decade, laser-based methods have emerged as a revolutionary approach toward the synthesis of graphene-based materials using various precursors, including silicon carbide,^11,12^ graphene oxide,^13−15^ various polymers,^16,17^ biomass,^18,19^ and the transformation of sp^3^ carbons to sp^2^ networks.^20^ Lasers play a transformative role in manufacturing, combining precision with scalability. Their digital control allows intricate designs to be executed with unparalleled accuracy, making them ideal for both detailed prototypes and large-scale production. Furthermore, the adaptability of laser parameters ensures versatility across a range of materials and nanostructures,^21,22^ paving the way for innovations in diverse industries and applications.^23^ In the case of laser-induced graphitization of polyimide (PI), which is the most commonly selected polymer precursor, the vast majority of works rely on the use of a CO_2_ laser to benefit from the high absorbance of the precursor at 10.6 μm. A major shortcoming accompanying this particular method of laser-assisted graphene synthesis on PI foils is that the laser-grown graphene is adhered on the PI substrate. For any application, graphene should be tested along with PI as a substrate, which severely limits potential use in real life products. To overcome this shortcoming, a manual transfer of graphene to other substrates (acceptor substrate) is required. In certain cases, complex processes have been undertaken, employing mold casting onto the irradiated PI followed by peeling the graphene off the PI substrate after the solidification of the acceptor substrate.^2,24,25^ However, such transfer methods lack efficacy and universality because a very limited class of acceptor substrates can be mold-casted. Further, this complex postsynthesis processing can have adverse effects on the quality and mechanical properties of the transferred graphene films.

To overcome the above limitations, we have recently established a novel method, which employs a simple and scalable process to prepare high-purity 3D-graphene scaffolds composed of few-layer turbostratically arranged graphene layers. This takes place by irradiating a carbon precursor (selected among a wide class of materials), employing laser-assisted explosive synthesis and transfer of graphene flakes (LEST).^26^ The precursor film (donor) is placed at a certain distance from the substrate (acceptor) onto which the graphene film is deposited. The method is versatile as it can operate with a combination of precursors, hence resulting in graphene nanohybrids, for example, graphene decorated with inorganic nanoparticles or heteroatom-doped graphene. Typical substrates that have been used include soft polymers, textiles, various metals, glass, ceramics, Si, and so on. Graphene-based materials produced by the LEST method result in graphene of very high purity (C/O ratio of ∼30), and high sp^2^ content of turbostratic stacking, which endows the film with a low sheet resistance. An additional merit of the LEST method is that a wide variety of carbon sources can be used, including biomass-derived products by appropriately tuning the irradiation conditions. This advantage provides independence from the ubiquitous use of PI films.

In the current work, we employ a modified version of the LEST method for graphene synthesis, transfer, and patterning in which the laser propagation direction has deliberately been chosen to be properly inclined, departing from the perpendicular incidence onto the acceptor substrate surface. A potential weakness of the LEST process using a perpendicular laser propagation geometry is that the trace of the transmitted (through the precursor material) laser beam falls within with the area of the deposited graphene film. Depending on the laser fluence, this might result in the (partial) ablation of the already deposited graphene flakes, also causing undesired laser-induced heating of the underlying precursor substrate. Thus, for heat-sensitive acceptor substrates (flexible electronics applications) it is essential to mitigate the effect of laser-induced ablation of the graphene film. We show in the current study that this can be achieved through the combined effect of an increased precursor–acceptor substrate distance and the inclination to the laser beam axis. For the purpose of this work, we use a slight inclination of 10–15° and examine the influence of the precursor–acceptor substrate distance on the electrical properties of the deposited graphene films. After exploring the effect of the donor–acceptor separation distance for different flexible acceptor substrates, the homogeneous graphene-like coating on a typical polymeric substrate, namely, polytetrafluoroethylene (PTFE), has been achieved through patterning the graphene deposition in the form of an array of interdigitated supercapacitor electrodes. The performance of such planar, interdigitated, binder-free supercapacitor devices was found to be superior to other relevant laser-based devices, showcasing the high prospects of this simultaneous synthesis and transfer of graphene approach. We emphasize that our prior research focused on optimizing the synthesized graphene-like structures.^26^ The present study introduces a new dimension to the fabrication process, offering capabilities not readily achievable with other laser-based techniques. Consequently, the advancements highlighted in this work pave the way for producing devices that surpass the performance of their existing counterparts made through laser-assisted methods. Specifically, we demonstrate that, by adjusting certain fabrication parameters (which do not affect the quality of graphene), we can substantially enhance the electrical attributes and facilitate electrode patterning, thereby improving the original LEST method.

## 2 Materials and Methods

### 2.1 Optimization of Off-Axis Deposition of Graphene-like Films

The preparation of the graphene-based films was achieved using the LEST method, which has been described in detail elsewhere.^26,27^ In brief, a millisecond pulse Nd:YAG (1064 nm) laser was used to irradiate a PI foil (DuPont Kapton HN), which served as the donor precursor. It has been demonstrated that this process enables the transfer of few-layer graphene flakes of turbostratic structure, directly onto the acceptor substrate. The different acceptor substrates used in the current study include polytetrafluoroethylene (PTFE), polydimethylsiloxane (PDMS), paper, cork, and cotton fabric. The lasing parameters used in this work include a laser fluence of ∼74 J cm^–2^ as well as a pulse width and spot size of 1.5 ms and 1.4 mm, respectively. To allow the formation of homogeneous coatings, a spot size overlap of ∼50% was used. According to our previous studies, decomposition of polyimide with a laser fluence of 74 J cm^–2^ yields graphene structures of the highest quality.^26,27^ In the current work, the LEST method is further optimized by addressing the “transfer step” of the process by changing the irradiation geometry. The laser fluence, which determines the “synthesis step” of the process and the initial conditions governing the transport of the synthesized flakes, is kept fixed at 74 J cm^–2^. The incident laser beam was placed off-axis in relation to the direction perpendicular to the substrate plane by ∼15°. The mass loading achieved is ∼0.5 mg cm^–2^.

### 2.2 Physicochemical Characterization

The distance between the precursor and the acceptor substrate was varied from contact configuration up to a 10 mm separation. The preferred distance for each acceptor substrate was optimized in terms of the (lowest) sheet resistance of the graphene films. The sheet resistance was measured using a four-point probe system (Ossila). The graphene porous films were prepared in a rectangular shape with dimensions of ca. 1 × 2 cm^2^ for the case of the flexible substrates, and 1 × 1 cm^2^ for the case of the Si substrates. Raman spectra were recorded with a micro-Raman system (Jobin-Yvon T-64000) equipped with a 514.5 nm laser line. An objective of 50× magnification was used, whereas spectra were calibrated with respect to the ∼520 cm^–1^ band of crystalline Si. The morphology of the graphene-like films was examined using a field-emission scanning electron microscope (Zeiss SUPRA 35VP) operating at 20 kV. The surface chemistry of the graphene films was investigated with X-ray photoelectron spectroscopy (XPS), conducted at ultrahigh vacuum (5 × 10^–10^ mbar). XP spectra were recorded using the Mg Kα (1253.6 eV) source, whereas acquisition and fitting of the spectra were performed with the commercially available software SpecsLab Prodigy (Specs GmbH, Berlin). The percentage contribution of the individual chemical states is based on the peak areas. The sp^2^ carbon content was calculated by adding the main sp^2^ peak at 284.4 eV and its shakeup feature at 290.7 eV.^28^ A stylus XP-1 Ambios Technology profilometer was used to assess the thickness of the porous graphene film deposited on a flat Si substrate.

### 2.3 Preparation of Interdigitated Electrodes and Evaluation of Supercapacitor Performance

The interdigitated capacitor electrodes were fabricated onto PTFE (100 μm thickness) using two consecutive laser processing steps. At first, a continuous graphene film was prepared by LEST, separating the PI–PTFE pair by a distance of 5 mm. Then, the same laser was used to selectively remove parts of the film, turning the continuous film into two patterned arrays of electrode fingers. The lasing parameters for the ablation processing step were as follows: spot size diameter of ∼0.2 mm, pulse width of 0.4 ms, and laser fluence of 30 J cm^–2^. For the laser setup used, the laser spot size and pulse width were set to their minimum values of 0.2 mm and 0.4 ms, respectively. This choice is critical for minimizing the gap between the two electrodes. Additionally, these parameters are optimal for reducing the size of the heat-affected zone (HAZ), which is linked to the thermal effects resulting from longer laser pulses, such as those produced by the laser setup currently in use. By application of the previously mentioned parameters for spot size and pulse width, the laser fluence was incrementally increased to a level (30 J cm^–2^) at which the LEST-deposited graphene could be effectively removed with a single laser pulse. A pulse overlapping of ∼75% was used in the ablation processing step, to ensure almost complete removal of the graphene flakes, hence avoiding short-circuit paths between the two electrodes. The active geometrical area of the supercapacitor (finger array including the separating trenches) was ∼1.5 × 1 cm^2^, and each electrode comprised four fingers (the width of each finger is ∼1.7 mm). Following the electrode fabrication, carbon cement (EM-Tec C38) was applied to the branches of the electrodes, to electrically connect them with Cu tape (EM-Tec). Kapton tape was placed on top of the contacts to protect them from the electrolyte.

The gel electrolyte was prepared as follows: 10 mL of 3D H_2_O was heated at 80 °C, while 1 g of PVA (M_W_ 9,000–10,000, 80% hydrolyzed) was slowly added under continuous stirring. After complete dissolution of the PVA, 1 mL of concentrated H_2_SO_4_ was added into the PVA solution. The aqueous PVA/H_2_SO_4_ electrolyte was cooled to room temperature and then was drop-casted onto the electrode finger arrays. The device was soaked in the electrolyte and was heated at 60 °C for 15 min. Then, it was placed inside a vacuum desiccator for 1 day to remove the trapped air introduced during stirring. Finally, the device was stored at ambient conditions for six days before its electrochemical characterization, to achieve the proper gelation of the electrolyte.

The interdigitated supercapacitor was assessed using cyclic voltammetry (CV), galvanostatic charging/discharging (GCD), and electrochemical impedance spectroscopy (EIS) using an electrochemical workstation (VersaSTAT 4, AMETEK SI, USA). The EIS measurements were conducted in the frequency range 100 kHz–0.01 Hz using an amplitude of 5 mV. The cycling stability was evaluated with a CTS-LAB system (BaSyTec GmbH, Asselfingen, Germany). To examine whether the supercapacitor can be operational under bending conditions, it was folded along its long axis around a high-curvature cylinder (9 mm diameter), which roughly corresponds to an angle of ∼180°. The areal capacitance of the device (mF cm^–2^) was calculated using the following equation^29^
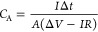
1where *I* is the applied current, Δ*t* is the discharging time, *A* is the area comprised by the two arrays of the fingers and the gap that separates them (1.5 cm^2^), and (Δ*V– IR*) is the voltage window minus the *IR* voltage drop in the beginning of the discharge curve.

The areal capacity (μAh cm^–2^) was calculated using the following formula:
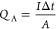
2

As the galvanostatic discharge curves deviate from linearity, the following equations were used to estimate the areal energy and power density:
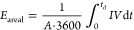
3
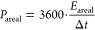
4

## 3 Results and Discussion

**Figure 1:**
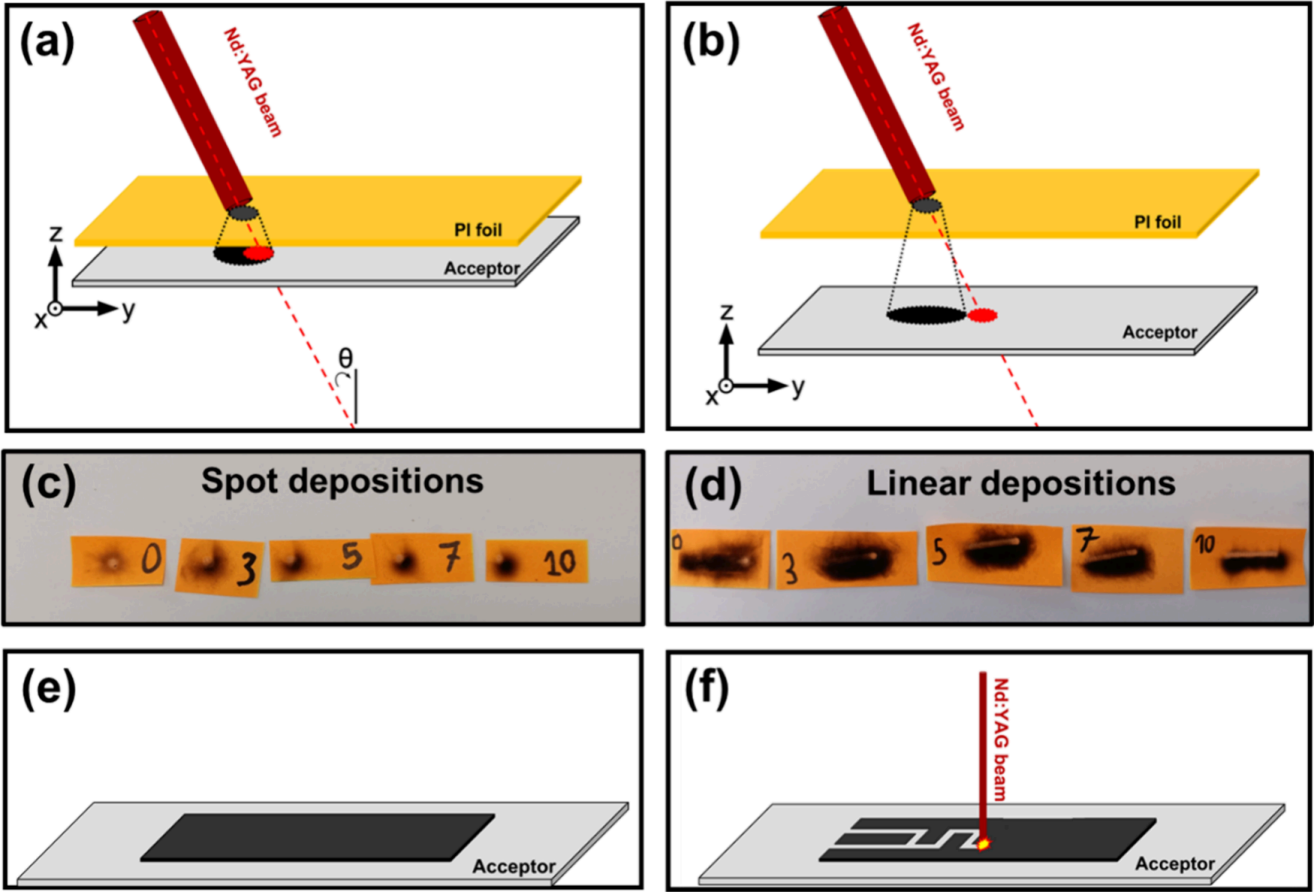
Graphene film deposition using the LEST method. (a) Small inclination angle and small donor–acceptor distance. (b) Large inclination angle and large donor–acceptor distance. Traces of (c) the spot (single lase) and (d) linear (scanning) depositions on colored paper. The traces correspond to various distances, namely, 0, 3, 5, 7, and 10 mm, and an inclination angle of ∼15°. (e, f) Schematics of the selective laser-assisted ablation to transform continuous LEST graphene films to patterned electrodes.

As has been detailed elsewhere, the LEST process is effective in decomposing a carbon precursor toward the formation of porous graphene-like networks.^26,27^ A strong requirement is that the precursor’s violent decomposition process should entail the production of propelling gases, providing enough momentum to the products (graphene flakes), for their transfer and high impact deposition on the acceptor substrate. According to the irradiation geometry of Figure 1, the ejected graphene flakes are inscribed within a cone-shaped volume.^30,31^ The adherence of the graphene film onto the acceptor substrate depends on the thermodynamics of the interaction between the graphene particles impinging on the substrate. This interaction can vary significantly in terms of many parameters (laser fluence, distance, nature of the substrate, temperature of the graphene particle, type of precursors, and so on). While the surface of sensitive substrates (polymers, paper, textiles, etc.) could be slightly modified/molded if the temperature of the deposited graphene particles exceeds their respective “softening” temperature, hard substrates such as ceramics, Si, or refractory metals (such as Mo) are not affected by the temperature of the deposited particles. Apart from the possible role of the deposited particles’ temperature in adhesion, the macroscopic film adhesion is likely affected by the roughness of the acceptor substrate. It is worth noting that the roughness of the substrate could be significantly influenced by the laser beam if the precursor absorbed at the laser wavelength. Therefore, the adhesion of LEST-graphene on a broad variety of acceptor substrates is likely due to their intermolecular interactions. For the particular case where the precursor film is a PI foil, an important condition for the process to occur is that the laser beam should propagate throughout the whole thickness of the precursor so that the precursor’s surface facing the acceptor substrate is the one that provides the graphene flakes to the substrate.

In the course of graphene film deposition using the LEST configuration, the laser beam is not completely absorbed by the donor (PI) film. Hence, in the case of perpendicular beam irradiation, a fraction of the laser pulse transmitted through the donor impinges on the acceptor substrate. In this geometry, the beam trace coincides with the deposited graphene material, which causes partial ablation of the graphene film and results in a corona-shaped deposition. Despite this shortcoming of the perpendicular irradiation geometry, these corona-shaped deposits can percolate into a continuous coating during a laser-beam scanning process provided that the spatial overlap of the pulses is suitably selected. If the propagation of the laser beam is slightly inclined to attain an off-axis configuration in relation to the perpendicular direction and when the separation gap between the carbon source and acceptor substrate is properly selected, the penetrating beam trace will gradually move out of the area where the graphene film is deposited (Figure 1a,b). An alternative method for displacing the trace of the transmitted laser beam out of the LEST graphene film would be to increase the inclination angle and keep the distance fixed. However, one should consider that a more inclined laser beam is tangled with operational safety in a roll-to-roll configuration, where the LEST is currently under development. Also, a larger deviation from the perpendicular direction could introduce technical intricacies in the deposition of graphene on large-scale applications compatible with flexible electronics technologies. In addition, larger angles engender a severe ellipticity of the laser beam cross section on the precursor substrate. Hence, a readjustment of the laser beam energy and the pulse overlap either in the X or in the Y axis must be performed for every change in the incidence angle. Trials have shown that keeping a low inclination and adjusting the distance are the most technically feasible way of displacing the penetrating laser beam trace from the deposited LEST graphene film. It should be noted that the deposited graphene structures span across an area that is larger than that defined by the laser beam spot size (see Figure 1c). Hence, the single-laser scan deposition of a pattern using our laser setup will inevitably suffer from low spatial resolution. As depicted in Figure 1e,f, an alternative solution to micropatterning with controlled spatial resolution (determined by the specific laser beam spot size) involves the use of second laser scan to selectively remove pre-deposited matter.

**Figure 2:**
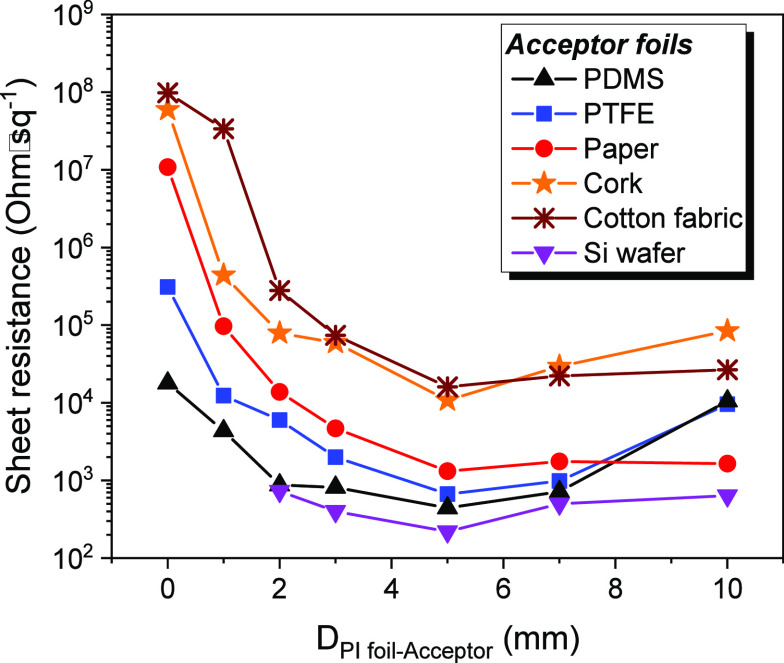
Dependence of the sheet resistance of the graphene-like depositions on the distance between the polyimide foil carbon source and the acceptor substrate for the cases of PDMS, PTFE, paper, cork, cotton fabric, and Si.

The proposed LEST process has been used to deposit graphene coatings on different flexible substrates, which were used as electrodes for the fabrication of flexible microsupercapacitors, as well as on nonflexible inorganic Si substrates, which may be of high importance for different applications. As these flexible substrates are electronically insulating and there is no current collector involved in the supercapacitor configuration used, the electronic conductivity of the transferred graphene films should be as high as possible. Therefore, the optimization of the deposition parameters related to the distance between the precursor and the flexible substrate was based on the minimization of the sheet resistance *R*_s_, while all other irradiation parameters were kept fixed. Figure 2 shows the influence of the donor (PI)–acceptor (flexible substrate) distance on *R*_s_ for various types of substrates. All curves show the same trend, exhibiting minimum *R*_s_ values at a distance (gap) of ∼5 mm. Paper exhibits a rather flat curve of *R*_s_ at higher gaps, which indicates a high potential of preparing conductive paper using dry deposition methods. This is an interesting finding because paper has recently attracted interest in flexible electronics.^32^

As the gap distance increases, there are two competing factors determining the observed trend in *R*_s_: (i) The trace of the beam that penetrates the PI foil (red spot in Figure 1a), which is responsible for the partial ablation of the deposited film, shifts away from the area where graphene flakes have just been deposited (black spot in Figure 1a). (ii) At the same time, increasing the distance that the graphene particles have to travel will result to fewer successfully adhered particles. As expected, if there is no gap separation (donor and acceptor in contact), *R*_s_ attains its highest value because the central region of the deposited film is partially removed.

**Figure 3:**
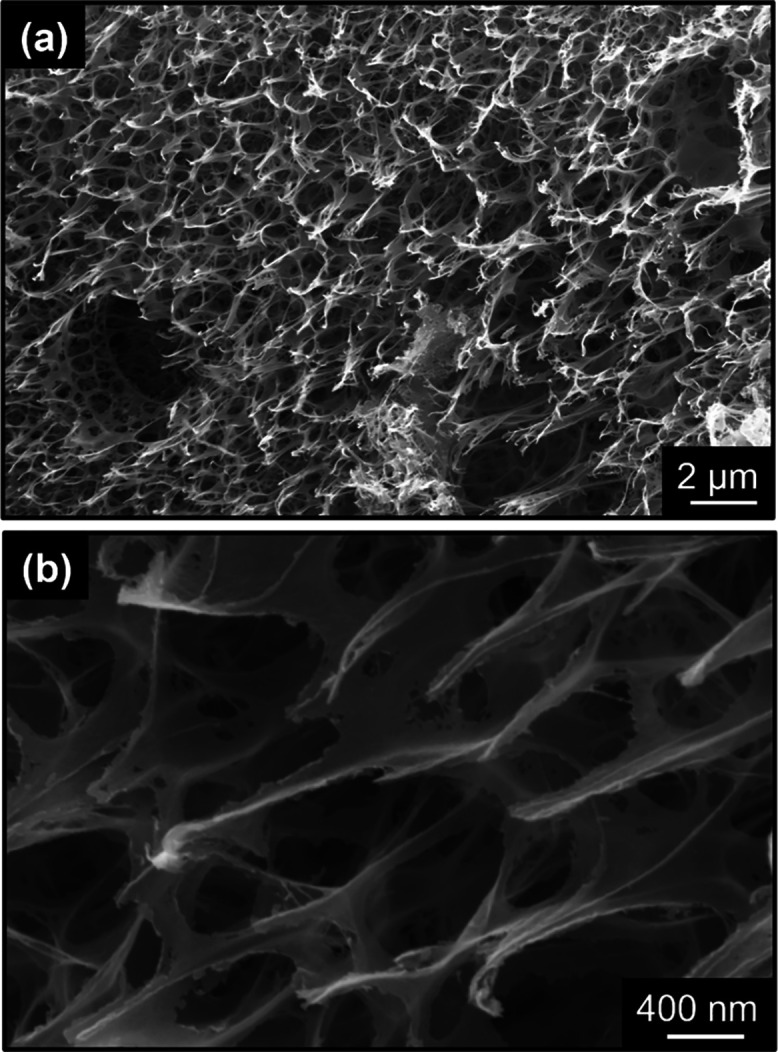
Scanning electron microscopy images at (a) low and (b) high magnifications of LEST-5-PTFE.

Based on the findings discussed above for the properties of the LEST graphene-coated flexible substrates, it seems that PTFE and PDMS are the substrates that offer the lowest *R*_s_ values. Nonflexible Si is the substrate providing the lowest sheet resistance of 220 ohm sq^–1^, which might be assigned to the lower roughness of the Si surface or/and to the much lower thermal sensitivity of Si in comparison to the other organic substrates. Interestingly, depositing LEST graphene on the diced Si wafers with a (precursor) polyimide–(substrate) Si distance lower than 2 mm resulted in the disintegration of the Si wafers. This arises from the mechanical stresses that follow the rapid and nonhomogeneous temperature rise when the graphene deposition and the transmitted laser beam trace overlapping is higher. While the resistance of LEST-graphene coated on PDMS is slightly lower, the adhesion of the graphene film formed is superior for the PTFE substrate. This is because PDMS (unlike PTFE) suffers from carbonization at 1064 nm; hence, its surface is decomposed after laser irradiation (see Figure S1). The decomposition of the PDMS results in the formation of loosely bound particles (debris) that contaminate the surface of the PDMS. This means that the LEST graphene flakes do not properly attach onto a clean PDMS surface. The inferior adhesion of the LEST graphene flakes on PDMS in comparison to the PTFE is observed in Figure S2. The graphene films prepared using a gap distance of 5 mm (referred to as LEST-5-PTFE) was selected for the fabrication of flexible supercapacitors. The morphology of the LEST-5-PTFE deposited graphene film is presented in Figure 3. Its porous texture results from the rapid outgassing, which follows the high temperature that is locally reached during laser irradiation and material decomposition. Based on transmission electron microscopy and N_2_ physisorption analysis from our prior research, it was determined that the porous structures consist of few-layered turbostratic graphene stacks featuring an expanded interlayer spacing. These structures are predominantly macroporous, exhibiting a specific surface area of approximately 120 m^2^ g^–1^.^26^

**Figure 4:**
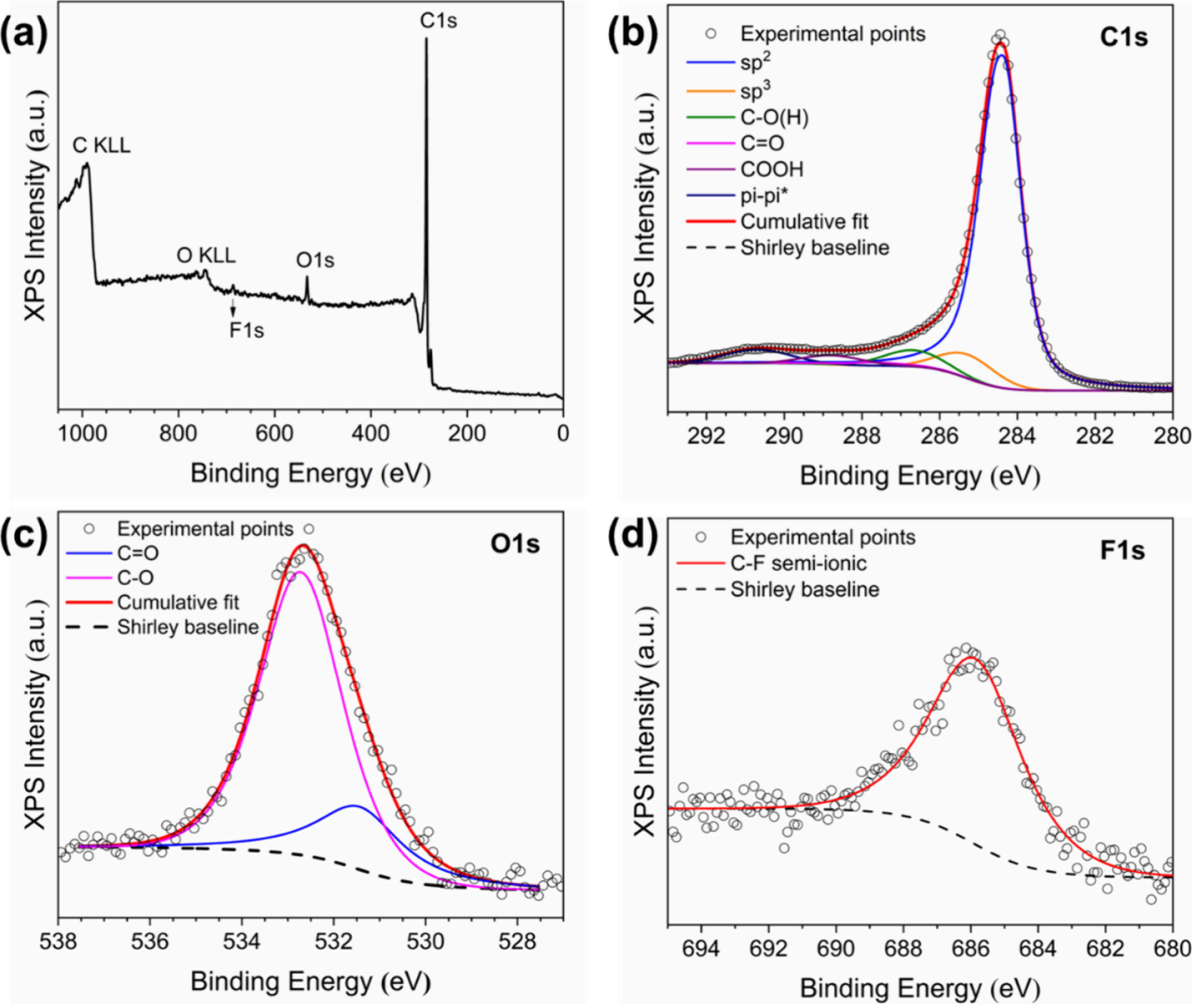
(a) Wide-scan X-ray photoelectron spectrum, (b, c) deconvoluted C 1s, O 1s photoemission peaks, and (d) fitted F 1s photoemission peak of LEST-5-PTFE.

The surface chemistry of LEST-5-PTFE was examined with XPS, as shown in Figure 4a, where quantitative analysis of the spectra resulted in the following element concentrations: C (97.0 at. %), O (2.7 at. %), and F (0.3 at. %) The C 1s photoemission peak (Figure 4b) was analyzed in the following components, sp^2^, sp^3^, C–O, C=O, and COOH. The π–π* satellite peak is also resolved at 290.7 eV. The binding energies and the percentage contributions of the above chemical states are listed in Table 1.

**Table 1:** Binding Energies and Concentration of Carbon Species in LEST-5-PTFE

C–C sp^2^	C–C sp^3^	C–O	C=O	COOH	π–π*
284.4 eV	285.4 eV	286.6 eV	287.7 eV	288.8 eV	290.7 eV
84.7%	6.5%	4.8%	1.4%	2.6%	

The O 1s has been deconvoluted into two components, namely, C–O at 532.7 eV and C=O at 531.5 eV (Figure 4c). The presence of a very small percentage of F atoms arises from the substrate. However, the F 1s binding energy of 686.0 eV does not correspond to the characteristic unit C–F_2_ of PTFE, which lies at 689.0 eV.^33^ The binding energy indicates the formation of C–F semi-ionic bonding.^34^ As has been explained above, a fraction of the laser pulse interacts with the substrate, which can be partly ablated and thermally decomposed, resulting in this very low but detectable F content on graphene surface (Figure 4d).

Such high ratios for C/O (∼36) and sp^2^/sp^3^ (∼13) typically correspond to materials with remarkably high electronic conductivity.^16,17^ However, no matter how high the conductivity for an individual flake might be, the macroscopically measured sheet resistance may increase by orders of magnitude if the junction resistance between flake or particle boundaries is high.^35^

**Figure 5:**
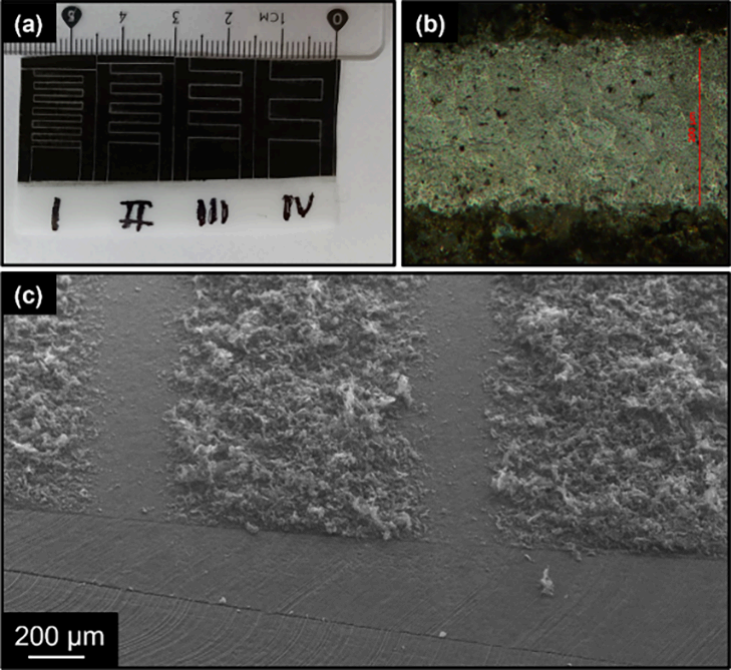
(a) Laser-patterned interdigitated electrodes on PTFE, (b) optical microscopy image, and (c) scanning electron microscopy image of the ablated path.

To fabricate an in-plane supercapacitor, a second lasing process was used to selectively ablate the graphene film, forming trenches that separate two neighboring fingers of the interdigitated electrodes, as shown in Figure 5. The geometrical aspect ratio of the electrodes’ fingers can be adjusted by programming the motorized *x*–*y* stage. Figure 5a demonstrates the final patterns for the case of six, four, three, and two fingers for each electrode. Figure 5b,c illustrates an optical microscope image and a SEM image of the ∼200 μm wide trench, showing that there is no conductive path, which could provide a short circuit between the two electrodes. As can be seen from the SEM image of Figure S3a and the surface profile of Figure S3b, the thickness of LEST graphene is about a few tens of microns, exhibiting quite strong variations. Repeated bending (100 times at a 180° angle) of the porous graphene depositions on PTFE led to the development of microcracks, as observed in Figure S4. These microcracks reduce the connectivity between individual graphene structures, resulting in an increase of approximately 24% in sheet resistance from 669 ohm sq^–1^ in the as-prepared film to 831 ohm sq^–1^. Figures S3 and S4a both reveal that the thickness of the LEST-graphene depositions varies across the deposited area. This is evidenced by the presence of a significant fraction of loosely bound structures that extend outward from the tightly adhered porous graphene network. As shown in Figure S5, when Scotch tape is applied to the patterned LEST-graphene electrodes, it removes the upper portion of the graphene film, which adheres to the sticky surface of the tape. However, a significant portion of the graphene film still remains attached to the PTFE support substrate. This outcome is particularly encouraging given that the LEST film is created through a dry process without any binder material or calendaring process, which are commonly employed in electrode fabrication. It is also important to highlight that this adhesion test is considerably more violent compared to the typical bending encountered by a supercapacitor, which often utilizes gel electrolyte and may be encased in a protective shell that helps compress the device’s components together. It should be mentioned that one additional reason for choosing a PTFE over a PDMS (apart from the inferior adhesion of LEST-graphene on PDMS) arises from the laser absorbance of the latter polymer (see Figure S1) as laser-based electrode patterning will leave carbon residues that may short-circuit the interdigitated electrodes.

**Figure 6:**
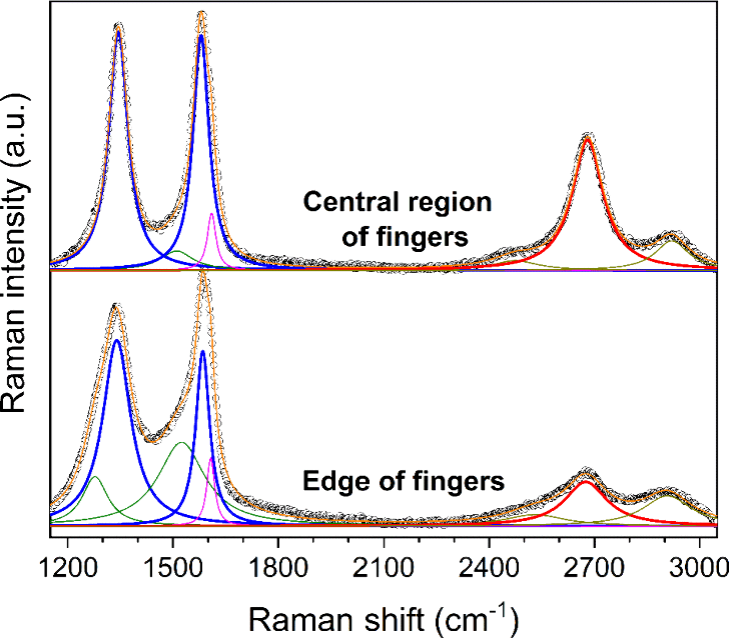
Comparison of the Raman spectra acquired far and near the laser-scribed path.

At this point, it is instructive to examine whether the laser patterning changes the carbon quality near the ablated path. Representative Raman spectra provided in Figure 6 support that the graphene structures near the trench are more defected than those in the central finger part. In the central (unaffected) region of the fingers, the Raman spectra of carbon present features that testify to the presence of high-quality graphene-like structures. Namely, the D (∼1345 cm^–1^), G (∼1580 cm^–1^), and 2D (2680 cm^–1^) bands of carbon are fairly sharp, indicating that the structures are highly crystalline, whereas the 2D band is intense and can be fitted with a single Lorentzian curve, which manifests the absence of Bernal stacking, and points toward the presence of rotational defects among the graphene layers.^36^ The intensity of the D band denotes an appreciable fraction of non-sp^2^ atoms (defects), which is reasonably expected for such 3D porous structures. Defects may include, but are not limited to, adatoms, edge, and curvature effects. According to an analysis by Ferrari and Robertson, the position of the G band denotes an sp^2^ hybridized network.^37^ The features of the Raman spectra obtained at the edge of the fingers are evidently different from those acquired prior to ablation. First, the D/G band area ratio is larger, suggesting that the basal plane structure of graphene is more defected.^38^ Second, the interbands located within the range 1200–1600 cm^–1^ are much more prominent. These bands emerge in cases where the D band is intense and have been assigned to the finite size of crystallites.^37^ The interband centered at ∼1520 cm^–1^ is blue-shifted, which could imply a slight oxidation of the graphene-like structures near the ablated path.^39,40^ Lastly, the second order 2D band is suppressed due to the higher concentration of defects, which disturb the hexagonal network of graphene.^41^ The higher degree of defects in the carbon structures near the trench may result from the prolonged heat transfer that follows the long pulse widths (milliseconds) of the laser beam.

**Figure 7:**
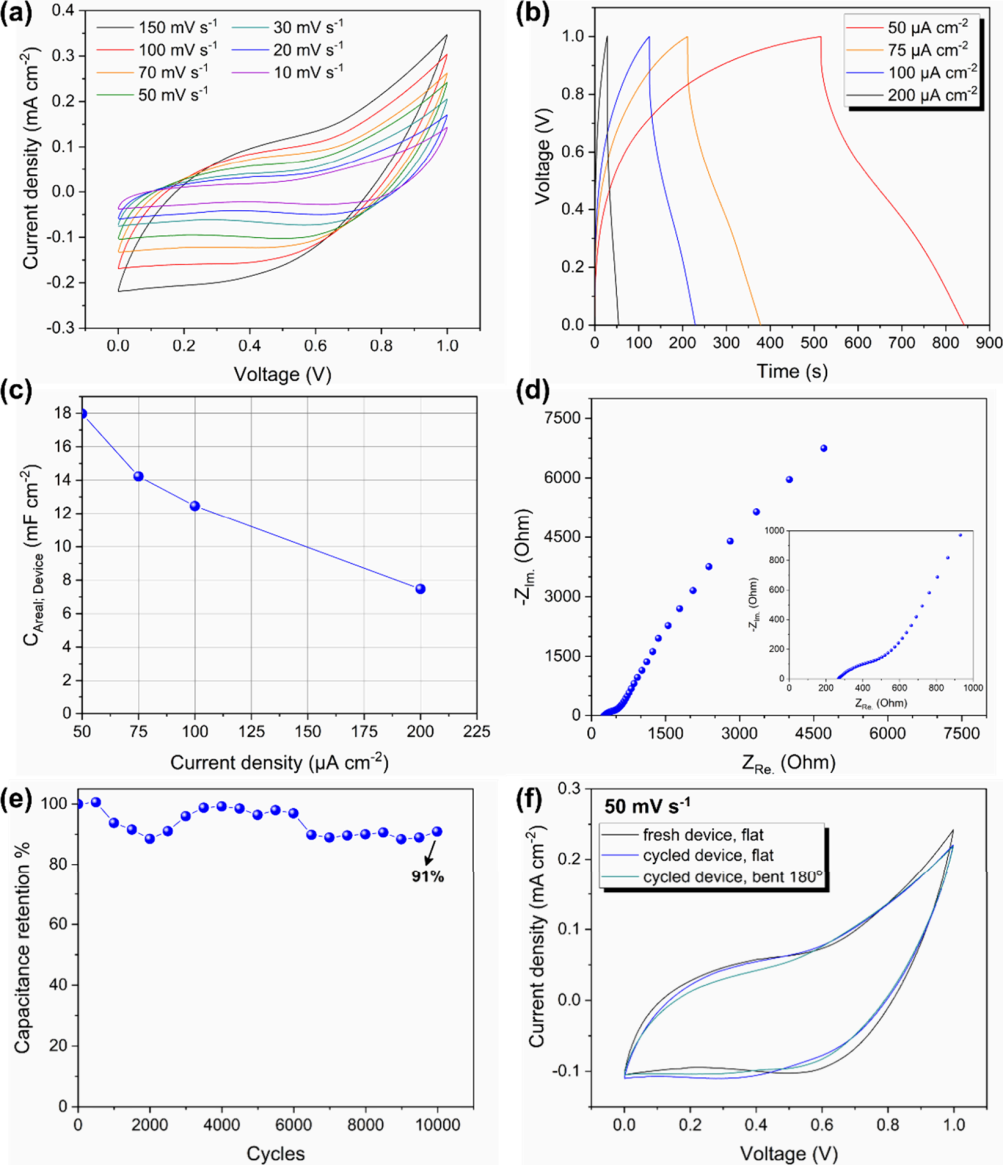
(a, b) CVs and GCD curves of the interdigitated supercapacitor, (c) areal capacitance of the device obtained from GCD curves, (d) Nyquist plot of the interdigitated supercapacitor (inset shows the higher frequency region), (e) cycling stability of the device (current density of 0.1 mA cm^–2^), and (f) CV comparison among the different states of the device (fresh and flat, cycled and flat, and cycled and bent).

The pattern II of Figure 5a, which contains four fingers per electrode, was selected to prepare the planar interdigitated supercapacitor device. The device was assessed in the operating voltage window between 0 and 1 V. Both the cyclic voltammograms (Figure 7a) and the galvanostatic charging–discharging curves (Figure 7b) reveal the characteristic behavior of an electric double layer capacitor, whereas pseudocapacitive contributions are also present. The latter can be observed from the considerable broadening of the CVs, which depart from a rectangular shape at voltages exceeding 0.5 V, and from the change in the slope of the discharge curves. Such behavior could manifest changes in the surface chemistry and morphology of the carbon structures (induced by cycling at the acidic electrolyte^40,42−44^ along with the occurrence of gas evolution reactions at voltages exceeding 0.7 V) or be due to the presence of heteroatoms.^45^ Oxygen and hydrogen evolution reactions (occurring concurrently at the negative and positive electrodes of the device) could entail the electrosorption/desorption of H_2_ at the negative electrode^46−48^ or the oxidation (and subsequent reduction) of carbon (oxygenated carbon) at the positive electrode.^48^ As will be mentioned in the following, despite the fact that the device is operating at a voltage window where gas evolution reactions occur, its performance during prolonged cycling remains essentially stable. The areal capacitance of the device derived from the GCD curves (Figure 7c) was measured to be ∼18.0 and ∼12.5 mF cm^–2^ at 0.05 and 0.10 mA cm^–2^ discharge currents, respectively. In terms of the areal discharge capacity, the above values correspond to ∼4.5 and ∼2.9 μΑh cm^–2^, whereas the areal energy (power) density is ∼1.9 μWh cm^–2^ (20.55 μW cm^–2^) and ∼1.1 μWh cm^–2^ (37.23 μW cm^–2^). These capacitance values are among the highest reported ones for interdigitated graphene supercapacitors, which are fabricated by laser-assisted methods.^16,49−56^ A comparison is presented in Table 2. The equivalent series resistance (ESR) was estimated to be ∼267 Οhm from the intersection of the impedance curve with the horizontal axis in the Nyquist plot, shown in Figure 7d. This ESR value is reasonable considering the gel electrolyte and the absence of a current collector and is comparable to ESR values that are commonly reported for similar supercapacitor configurations.^50,51,57^ As can be observed from the corresponding Bode plot shown in Figure S6, the characteristic relaxation time constant (τ) is 5.7 s. This value is comparable to that of previously reported solid-state supercapacitors utilizing graphene-like structures in their electrodes.^58,59^

**Table 2:** Comparison of the Areal Device Capacitance and Energy Density among Graphene-Based Interdigitated Supercapacitors Fabricated Using Laser-Assisted Processes

material/carbon precursor	transfer/method	C_A_ [mF cm^-2^]/E_A_ [μWh cm^-2^]	current density [mA cm^-2^]	scan rate [mV s^-1^]	ref
LEST graphene/PI	yes/laser	17.97/∼1.9	0.05		this work
graphene/PI	no	9.11/∼1.2	0.01		(49)
graphene/PI	no	3.9/∼0.3	0.2		(16)
graphene/GO	yes/manual	0.68/∼0.038		5	(52)
metal-doped graphene/PI	no	1.2/∼0.12		100	(53)
B-doped graphene/cork	no	4.67/∼0.65	0.10		(50)
N, S-co-doped graphene/GO	no	11.35/∼1.13	0.125		(51)
graphene/leaves	no	8.83/∼1.204	0.005		(54)
rGO/GO	yes/mold-cast and etching	1.94/0.172	0.01		(55)
graphene/PI	no	15.39/1.75	0.1		(56)

The interdigitated device seems to retain ∼91% of its initial capacitance after operating for 10,000 cycles at a current density of 0.10 mA cm^–2^ (see Figure 7e). The degradation may be related to the changes of the carbon surface during cycling in a way that is similar to previous reports on the corrosion of sp^2^ carbons at acidic pH.^44^ Finally, the mechanical stability of the device was tested against severe bending deformation. Figure 7f shows the comparison between the CVs of the supercapacitor in its as-prepared form after cycling for 10,000 cycles and after cycling and subjection to bending at ∼180°. The data reveal that, even under severe bending, the device performance is not substantially compromised. Indeed, the CV of the cycled-bent device is only ∼5% smaller than that of the cycled-flat device.

## Conclusions

In summary, we have used an alternative laser-based method to simultaneously synthesize and transfer porous 3D graphene-based structures, which are used for the fabrication of microflexible interdigitated supercapacitors. This approach offers a novel method to fabricate such devices, avoiding postsynthesis electrode processing, which usually compromises the quality of the grown graphene structures as in most previous reports. The current approach is a modification of a process we developed for graphene synthesis and transfer, namely, the laser-assisted explosive synthesis and transfer of graphene (LEST). This novel deposition method offers very high-quality graphene films composed of turbostratically arranged few-layer graphene with significantly higher C/O and sp^2^/sp^3^ ratios, that is, ∼36 and ∼13, respectively, compared to other laser-based approaches.

The modified method employs an alternative irradiation geometry, which is suitable for in situ depositing graphene films on highly sensitive substrates, typically used in flexible electronics. Inclining the incident laser beam with regard to the perpendicular direction, we avoid the direct exposure of the penetrating part of the beam with the just-deposited graphene film, hence evading unwanted ablation effects. Further, the modified geometry offers additional benefits for the film quality and the resultant sheet resistance.

Laser-patterning took place after deposition of the graphene film to prepare interdigitated electrodes. Microflexible interdigitated supercapacitors fabricated in this way were evaluated electrochemically. The analysis revealed an areal capacitance of ca. 18 mF cm^–2^ at 0.05 mA cm^–2^, which is appreciably higher than other capacitance values reported so far for interdigitated graphene-based supercapacitors prepared by laser-assisted methods. High retention levels after long cycling and resilience to bending effects demonstrate that the microflexible devices fabricated by the proposed laser-assisted process show high potential for transforming green and scalable manufacturing of flexible electronics and smart textiles.

## Supporting Information Available

PDMS directly irradiated by a 1064 nm laser source; as-prepared LEST-graphene films on PTFE and PDMS and air-blown LEST-graphene films on PTFE and PDMS; thickness profile of as-prepared LEST-5-Si; SEM images of LEST-5-PTFE after being subjected to 100 repetitions of bending at an angle of 180°; Scotch-tape peel-off adhesion test of laser-patterned LEST-5-PTFE; and Bode plot of the interdigitated supercapacitor (PDF)
