# Supplementary material for: Direct Graphene Deposition via a Modified Laser-Assisted Method for Interdigitated Microflexible Supercapacitors
Source: ACS Appl Nano Mater. 2024 Feb 9;7(4):3782–92. doi: 10.1021/acsanm.3c05387 (PMC11192044; doi:10.1021/acsanm.3c05387)
Supplement: Supplementary file 1 — an3c05387_si_001.pdf [file an3c05387_si_001.pdf]

Supporting Information

Direct Graphene Deposition via Modified Laser-Assisted Method for Interdigitated Micro-Flexible Supercapacitors

Nikolaos Samartzis[1,2,*], Michail Athanasiou[1], Labrini Sygellou[1], Spyros N. Yannopoulos[1,3,*]

[1] *Foundation for Research and Technology Hellas, Institute of Chemical Engineering Sciences (FORTH/ICE-HT), GR-26504, Patras, Greece*

[2] *Department of Physics, University of Patras, GR-26504, Patras, Greece*

[3] *Department of Chemistry, University of Patras, GR-26504, Patras, Greece*

*Corresponding authors: n.samartzis@iceht.forth.gr & sny@iceht.forth.gr

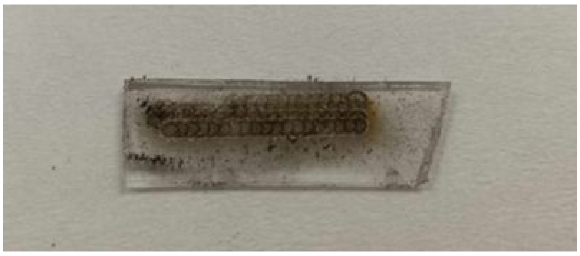

Figure S1: Optical image of PDMS directly irradiated by our 1064 nm laser source, using a laser fluence of 74 J cm^{-2}. The laser-induced carbonization of PDMS is evident, testifying that PDMS absorbs this laser wavelength.

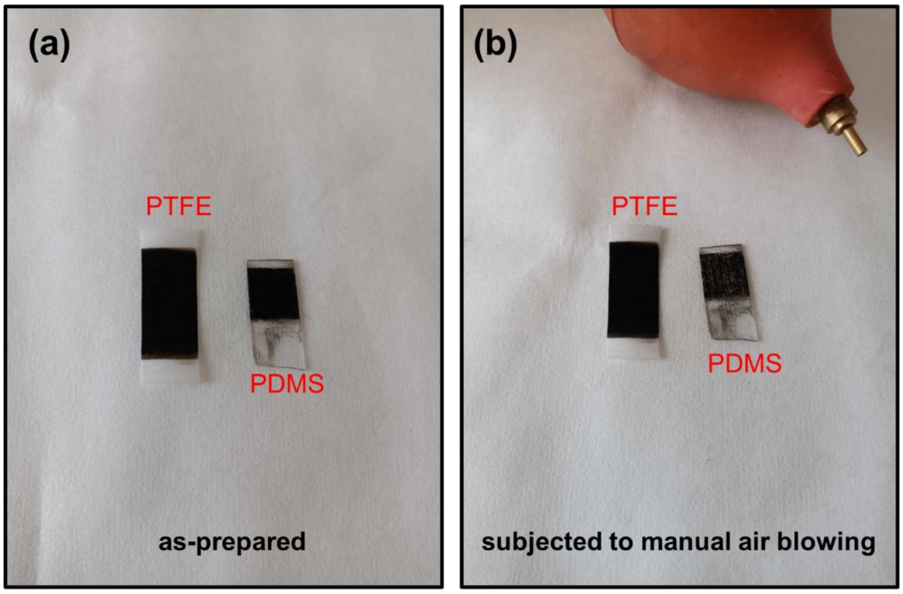

Figure S2: Optical images depicting (a) as-prepared LEST-graphene films on PTFE and PDMS, (b) air-blown LEST-graphene films on PTFE and PDMS.

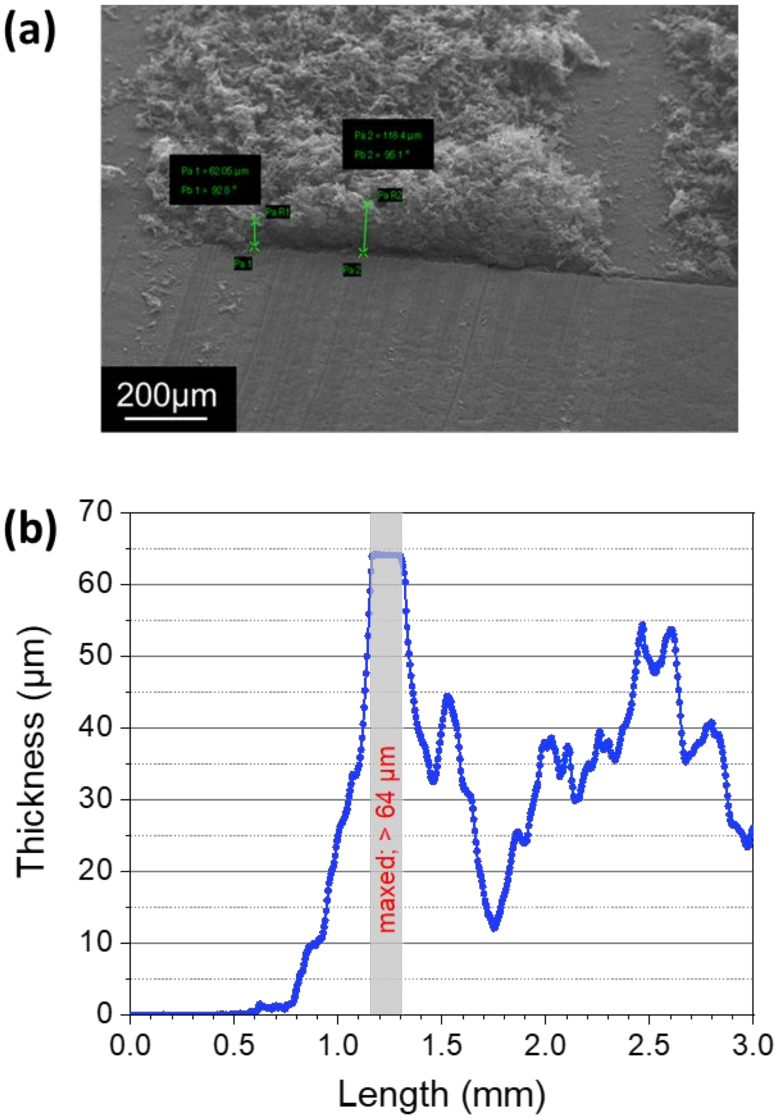

Figure S3: (a) Side view of LEST-5-PTFE after laser patterning, (b) thickness profile of as-prepared LEST-5-Si.

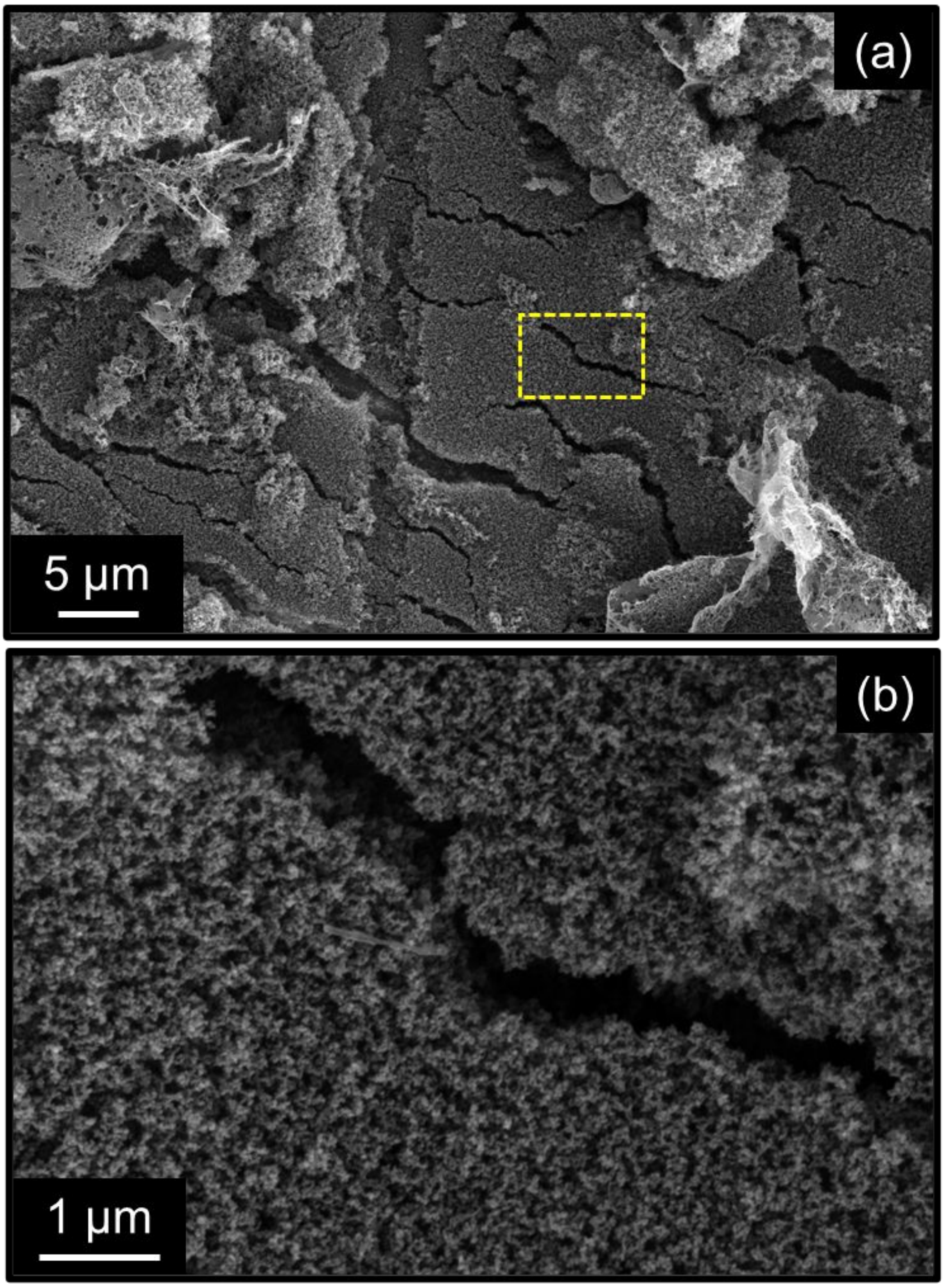

Figure S4: (a, b) SEM images of LEST-5-PTFE after being subjected to 100 repetitions of bending at an angle of 180°.

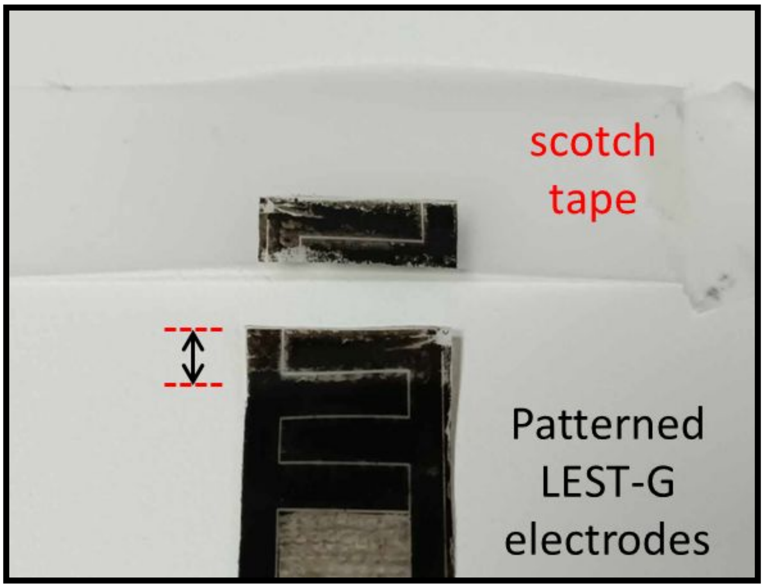

Figure S5: Scotch tape peel off adhesion test of laser patterned LEST-5-PTFE.

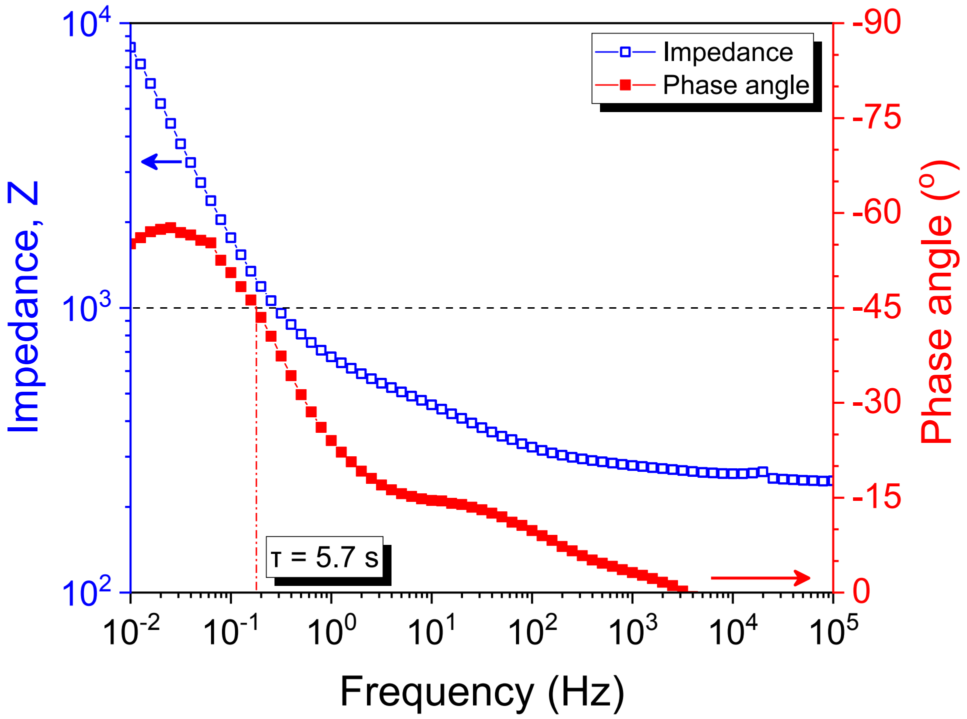

Figure S6: Bode plot of the interdigitated supercapacitor.
